# Amniocentesis in HIV Pregnant Women: 16 Years of Experience

**DOI:** 10.1155/2013/914272

**Published:** 2013-07-21

**Authors:** Mafalda Simões, Catarina Marques, Ana Gonçalves, Ana Paula Pereira, Joaquim Correia, João Castela, Cristina Guerreiro

**Affiliations:** ^1^Obstetrics/Gynecology, Maternidade Dr. Alfredo da Costa, Lisbon, Portugal; ^2^Gynecology Department, Maternidade Dr. Alfredo da Costa, Lisbon, Portugal; ^3^Prenatal Diagnosis Department, Maternidade Dr. Alfredo da Costa, Lisbon, Portugal; ^4^Pediatrics Department, Maternidade Dr. Alfredo da Costa, Lisbon, Portugal; ^5^Fetal Maternal Department, Maternidade Dr. Alfredo da Costa, Lisbon, Portugal

## Abstract

The iatrogenic risk of HIV vertical transmission, calculated in initial epidemiologic studies, seemed to counterindicate invasive prenatal diagnosis (PND) procedures. The implementation of highly active antiretroviral therapy (HAART) represented a turning point in PND management, owing to a rapid and effective reduction of maternal viral load (VL). In the present study, we identified cases of vertical transmission in HIV-infected pregnant women who did amniocentesis in the second trimester of pregnancy (*n* = 27), from 1996 to 2011. We divided our sample into Group A—women under HAART when submitted to amniocentesis (*n* = 20) and Group B—women without antiretroviral therapy before amniocentesis (*n* = 7). We had 1 case of vertical transmission in Group B. Preconceptional or early first trimester HIV serology is essential to avoid performing an amniocentesis without antiretroviral therapy or viral suppression. When there is an indication for amniocentesis in an HIV-infected pregnant woman, it should be done if the patient is on HAART and, if possible, when VL is undetectable. Nowadays, with combined first trimester screening test to select pregnancies with high risk of aneuploidies, advanced maternal age is a less frequent indication to perform PND invasive procedures, representing an outstanding gain in prenatal diagnosis of this population.

## 1. Introduction

The widespread use of highly active antiretroviral therapy (HAART) during the last decade has significantly reduced the rates of HIV mortality and disease progression [1]. The rate of vertical transmission in HIV-infected pregnant women on HAART is around 1-2%, being almost zero when associated with an elective cesarean delivery and avoidance of breastfeeding [2]. Simultaneously, there has been an increase in pregnancy rates among HIV-infected women, raising new problems and issues in prenatal diagnosis (PND), such as those concerning invasive procedures to diagnose chromosomal abnormalities (amniocentesis and chorionic villus sampling) [3]. The increase in the mean maternal age is a challenge in prenatal diagnosis, particularly in HIV-infected pregnant women. 

In the past, invasive procedures as amniocentesis were generally discouraged in HIV-infected pregnant women, due to increased risk of vertical transmission. The puncture of the uterine wall or placenta and lesions of the fetal skin or umbilical chord may all increase the fetal exposure to maternal virus [4]. Amniocentesis itself has potential morbidity, such as rupture of membranes, chorioamnionitis, or placental abruption, with consequent fetal loss or vertical transmission if gestation goes on [5]. 

Studies that analyze vertical transmission rates after amniocentesis have been scant, and existing data are limited. First studies report an increase in vertical transmission after procedures undertaken during the second or third trimesters of pregnancy [6–8]. However, during the pre-HAART era, amniocentesis was not performed in most of the centers, even if there was a medical/obstetrical indication, and therefore these studies include very small and heterogeneous samples. Since 2003, the reported risk of vertical transmission has markedly diminished [3, 9–11] because of the wide spread of antiretroviral therapy. Some centers, thus, started to offer amniocentesis during second trimester to HIV-infected pregnant women, whenever a strong indication (genetic or infectious) exists. These studies have reported no cases of vertical transmission after invasive procedures among HIV-infected women treated with HAART.

According to the British Guidelines [11], for women who have started HAART but whose viral load is not yet undetectable, it may be advisable to delay the amniocentesis until the maternal viral load is undetectable if at all possible. In women not already taking HAART, administration of antiretroviral therapy to cover the procedure is advised. 

The aim of our study was to identify cases of vertical transmission in HIV-infected pregnant women who did second trimester amniocentesis in our hospital.

## 2. Methods

**Figure 1:**
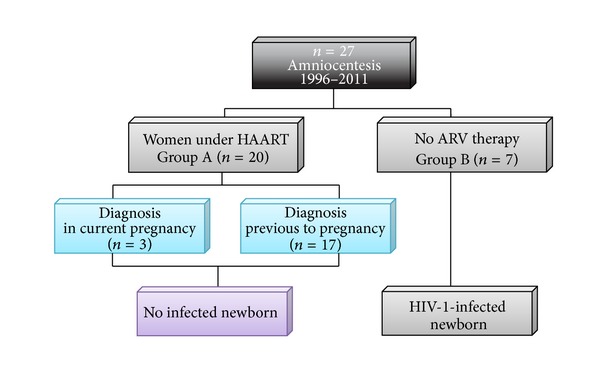
Summary of the study. HAART: highly active antiretroviral therapy; ARV: antiretroviral therapy.

We analysed amniocentesis (*n* = 27) performed in our institution from the observational cohort of HIV-infected pregnant women. The sample was obtained from the database, which included all HIV-infected pregnant woman who gave birth between 1996 and 2011 (*N* = 804). All clinical files were reviewed and data were collected in order to obtain demographic characteristics of the sample, risk factors associated with HIV infection (such as drug abuse), obstetrical variables such as parity, mode of delivery, obstetrical complications, indication of amniocentesis and gestational age when it was accomplished, HIV subtype and transmission category, antiretroviral regimen, viral load close to amniocentesis and close to labour, fetal karyotype, newborn data such as weight, antiretroviral prophylaxis regimen, and HIV DNA PCR. Our sample was divided into two subgroups: women under HAART when submitted to amniocentesis (Group A, *n* = 20) and women without antiretroviral therapy before amniocentesis (Group B, *n* = 7) (Figure 1). SPSS Version 17.0 was used to obtain statistical analysis of both groups and to compare differences in transmission rates among groups. The results were analyzed statistically using Chi-square. A *P* value below 0.05 was considered to indicate statistical significance.

This study was approved by the Ethical Committee of our hospital. 

## 3. Results

Between 1996 and 2011, amniocentesis was performed in 3.36% of our cohort (*N* = 804). 

### 3.1. Demographics

The mean maternal age of our study group was 37.7 years, and most of them were Caucasian and multiparous (Table 1). Sexual transmission of HIV was the main way of infection, and HIV 1 was the most frequent subtype (*n* = 21). In Group A, 2 women had double infection (HIV-1 and HIV-2) and other 2 were HIV-2 infected. The remaining women were HIV-1 infected. In Group B, 2 women had HIV-2 infection and 5 were HIV 1-infected. 

### 3.2. Characteristics of Amniocentesis

Advanced maternal age was the most frequent indication for amniocentesis. Gestational age at the time of amniocentesis was, in most cases, between 16 and 19 weeks (Table 2). Two cases of chromosomal abnormalities, both trisomy 21, were diagnosed. These two amniocentesis were performed, one for advanced maternal age and the other for augmented nuchal translucency.

In Group A, viral load (VL) at amniocentesis was undetectable in 11 cases (2 of these patients were HIV-2 infected), whereas in Group B viral load was unknown in all women. However, in 2 women of Group B, who started prenatal care only after amniocentesis, viral load was quantified subsequently one HIV-2-infected woman had undetectable viral load 4 weeks after the procedure and the other had 5.790 copies/mL 9 weeks after amniocentesis.

### 3.3. Pregnancy Outcome

The most frequent obstetrical complications in our study group were fetal growth restriction (FGR), preterm labour, and chronic hypertension (Table 3).

Elective cesarean after 37 weeks was the predominant mode of delivery in both groups. In Group A, VL determined close to labor was undetectable in 13 women, and in Group B it was undetectable in 4 women, the remaining 3 being unknown. 

Among the 27 newborns, only one case of HIV 1 infection was diagnosed, owing to Group B. It occurred in a patient with a diagnosis of HIV infection at 30 weeks of gestation (in 1998), who had already done amniocentesis at 16 weeks for primary CMV maternal infection. Primary care physician sent this patient at 9 weeks of gestation to our emergency department due to a rash, which was interpreted as an exanthem subitum. She was referred to our PND center and laboratory tests of CMV, Toxoplasmosis, Rubella, and Parvovirus B19 were requested. Since the patient had been referred from primary care, HIV serology had already been requested, and according to the patient, with pending results. Therefore, it was not requested in our tertiary care center. CMV results were positive for primary infection and the amniocentesis was planned. However, this patient was absent from medical care until 30 weeks, when she returned with a diagnosis of HIV infection (requested by her primary care physician), presumably due to a disease denial. Spontaneous labour began at 38 weeks and she had a vaginal delivery. She had done neither combination antiretroviral drug therapy nor AZT prophylaxis during pregnancy, as well as adequate prenatal care. AZT intravenous perfusion was done during labour. 

Among the 777 women who were not submitted to invasive procedures, there were 4 cases of vertical transmission (0.5%). When we compared this transmission rate with the transmission rate of 1/27–3.7% (all HIV pregnant women who underwent amniocentesis), no statistical significant difference was found (4/777 versus 1/27; *P* value = 0.4083; *χ* ^2^-test). Comparing the difference in transmission rate between Groups A and B, we obtained a *P* value of 0.5756 (0/20 versus 1/7; *χ* ^2^-test). 

### 3.4. Antiretroviral Therapy

**Figure 2:**
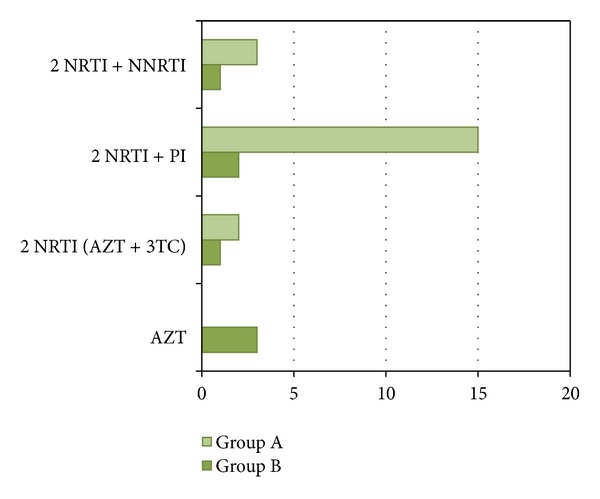
Antiretroviral therapy during pregnancy. AZT: zidovudine; 3TC: lamivudine; NRTI: nucleoside reverse transcriptase inhibitors; NNRTI: nonnucleoside reverse transcriptase inhibitors; PI: protease inhibitors.

Antiretroviral drug therapy in pregnancy is described in Figure 2. In Group B, therapy regimen was initiated in the second half of pregnancy, because of late HIV diagnosis (only after amniocentesis). Monotherapy prophylaxis with AZT was administrated to three pregnant women in Group B. 

## 4. Discussion

The absence of a control group and the small sample dimension raise some limitations to the statistical analysis in the present work. This is also the case in studies performed by other authors [3, 9–11].

In the particular case of vertical transmission in Group B, we cannot exclude the possibility of a primary HIV infection considering the rash as the initial manifestation of the disease. Primary HIV infection is a major risk factor for vertical transmission. Concomitantly, primary infection for CMV may reveal immunosuppression status of the patient, also contributing to vertical transmission. 

It is important to note that the long time span of our study includes a period when AZT monotherapy was considered to be the gold standard prophylaxis. Nowadays, regimens of HAART are recommended during pregnancy with two nucleoside reverse transcriptase inhibitors (NRTIs) and one protease inhibitor (PI) [10], allowing to achieve a higher efficacy and sustainability of viral suppression. According to Watts' review, the patient should have an optimized antiretroviral treatment and an undetectable viral load before undergoing an invasive procedure as amniocentesis [12]. In addition, López and Coll recommended that transplacental amniocentesis should always be avoided [13]. Therefore, it is important to propose a viral load determination before amniocentesis for patients with contamination risk and to postpone the invasive procedure in cases with positive results in order to obtain an undetectable viral load [14].

In addition, considering that this study involves a long time spam, it is also important to note that physicians have not always been sensitized to ask for an HIV test before performing PND invasive procedures. Also, advanced maternal age was the predominant indication for amniocentesis in our cohort. Nowadays, with combined first trimester screening test to select pregnancies with high risk of aneuploidies, advanced maternal age is a less frequent indication to perform PND invasive procedures. Among HIV-infected women this important screening test minimizes invasive procedures, representing an outstanding gain in prenatal diagnosis of this population.

Somigliana et al., in a multicenter study, showed no difference in transmission rate between mothers who underwent an invasive procedure and the control group (2/60 versus 12/712) [15]. In our study, we obtained an overall transmission rate of 4/777 (0.5%) in HIV pregnant women without invasive procedures, and a transmission rate of 1/27 (3.7%) in those who had an amniocentesis (4/777 versus 1/27; *P* value = 0.4083; *χ* ^2^-test). Therefore, due to the small sample dimension of our study and to the inadequate power for differences in transmission rates, there is no evidence that amniocentesis is associated with a higher transmission rate. This is also the case in other published series [8, 9, 11]. Moreover, when we compared transmission rates between our two Groups A and B, we found no statistical significant differences (0/20–0% versus 1/7–14.3%; *P* value = 0.5756; *χ* ^2^-test). 

In our opinion, invasive PND techniques should not be precluded to HIV-infected pregnant women at increased risk for fetal chromosomopathies. However, it is extremely important to adopt a selective attitude in these situations, informing and clarifying the patient about the risks related to the procedure. Besides the issue of perinatal transmission, it is essential to remember the complications inherent to invasive procedures, such as premature rupture of membranes, chorioamnionitis, and placental abruption, as previously stated. 

## 5. Conclusions

Preconceptional or early first trimester HIV screening is essential to avoid amniocentesis without antiretroviral therapy or viral suppression. When amniocentesis is indicated in HIV-infected pregnant women, it should be done after initiating a combination antiretroviral drug therapy and ideally when viral load is undetectable. The wide spread of combined prenatal screening tests has been particularly important in these patients, decreasing the need to perform invasive PND procedures. Although the size of our sample is limited, there was no case of vertical transmission among pregnant women with prenatal care, who have done amniocentesis on HAART. It would be extremely important to analyze wider results, in a multicentric study.

## Figures and Tables

**Table 1:** Demographic characteristics.

Demographics	Group A (*N*/%)	Group B (*N*/%)
Age (years)		
Mean 37.7		
<35	4/20%	1/14.3%
≥35	16/80%	6/85.7%
Race		
Caucasian	15/75%	4/57.1%
African	4/20%	3/42.9%
Indian	1/5%	—
Parity		
Nulliparous	5/25%	2/28.6%
Category of transmission		
Sexual	15/75%	7/100%
Substance abuse	4/20%	—
Transfusional	1/5%	—
Subtype of HIV		
HIV 1	16/80%	5/71.4%
HIV 2	2/10%	2/28.6%
Double infection (HIV-1 and HIV-2)	2/10%	—

**Table 2:** Characteristics of amniocentesis.

Characteristics of amniocentesis	Group A (*N*/%)	Group B (*N*/%)
Indication		
Advanced maternal age	15/75%	5/71.4%
Increased NT	2/10%	1/14.3%
CMV Infection	—	1/14.3%
Isoimmunization Rh	1/5%	—
Maternal genetic disease (cystic fibrosis)	1/5%	—
Malformation	1/5%	—
GA (weeks)		
13–15	—	1/14.3%
16–19	13/65%	4/57.1%
19–20	1/5%	—
≥20	6/30%	2/28.6%
Chromosomal abnormalities		
Trisomy 21	1/5%	1/14.3%
Viral load at the time of procedure	(copies/ mL)	(copies/ mL)
<50	11/55%	—
50–999	5/25%	—
1000–10000	1/5%	—
>10000	3/15%	—
Unknown	—	7/100%

NT: nuchal translucency; GA: gestational age.

**Table 3:** Pregnancy outcome.

Pregnancy outcome	Group A (*N*/%)	Group B (*N*/%)
Obstetrical complications		
Chronic hypertension	1/5%	1/14.3%
Foetal growth restriction	4/20%	—
Gestational diabetes	1/10%	1/14.3%
Preterm labour/PPROM	4/20%	—
Hydramnios	2/10%	—
Infection (CMV)	—	1/14.3%
Isoimmunization Rh	1/5%	
Mode of delivery		
Vaginal	4/20%	2/28.6%
Instrumental	1/5%	—
Elective cesarian	14/70%	5/71.4%
Cesarian during labour	1/5%	—
Viral load close to labour (copies/mL)		
<50	13/65%	3/42.9%
50–999	5/25%	
1000–10000	1/5%	
>10000	1/5%	
Unknown	—	4/57.1%

PPROM: preterm premature rupture of membranes; GA: gestational age.
